# *C. elegans* foraging as a model for understanding the neuronal basis of decision-making

**DOI:** 10.1007/s00018-024-05223-1

**Published:** 2024-06-08

**Authors:** Jessica A. Haley, Sreekanth H. Chalasani

**Affiliations:** 1 Neurosciences Graduate Program, University of California San Diego, La Jolla, CA 92093 USA; 2 Molecular Neurobiology Laboratory, Salk Institute for Biological Studies, La Jolla, CA 92037 USA

**Keywords:** Dietary choice, Patch-leaving, Exploration, Exploitation

## Abstract

Animals have evolved to seek, select, and exploit food sources in their environment. Collectively termed foraging, these ubiquitous behaviors are necessary for animal survival. As a foundation for understanding foraging, behavioral ecologists established early theoretical and mathematical frameworks which have been subsequently refined and supported by field and laboratory studies of foraging animals. These simple models sought to explain how animals decide which strategies to employ when locating food, what food items to consume, and when to explore the environment for new food sources. These foraging decisions involve integration of prior experience with multimodal sensory information about the animal’s current environment and internal state. We suggest that the nematode *Caenorhabditis elegans* is well-suited for a high-resolution analysis of complex goal-oriented behaviors such as foraging. We focus our discussion on behavioral studies highlighting *C. elegans* foraging on bacteria and summarize what is known about the underlying neuronal and molecular pathways. Broadly, we suggest that this simple model system can provide a mechanistic understanding of decision-making and present additional avenues for advancing our understanding of complex behavioral processes.

## Introduction

Foraging is a ubiquitous component of animal behavior as all animals require food and, for many, the search, pursuit, capture, and consumption of food is critical for survival and reproductive success. For decades, behavioral ecologists have sought to predict how foraging behaviors affect the fitness of an animal via quantitative modeling [1, 2]. Some of the most frequently-studied questions in foraging theory have probed an animal’s ability to: (1) locate food (food search), (2) choose between different food types (dietary choice), and (3) allocate time spent foraging within patches of food items (patch-leaving) [3–5]. Notably, the importance of each of these decisions to an animal’s survival is highly contextual. For example, while food search is often rate-limiting for foragers in habitats with sparsely distributed food (e.g., temperate ant communities [6, 7]), the critical decision for animals that encounter food frequently (e.g., honey bees [8, 9]) may be selecting which food types to include in their diet. In animals that both find and consume food quickly (e.g., numerous bird species [5, 10, 11]), the most important foraging decision may be when to leave a patch (i.e., a clump of food items) with depleting resources. In all cases, foraging is often a tradeoff between exploiting an environment for known resources and exploring elsewhere for potentially better opportunities [5, 12]. These foraging decisions are thus goal-directed behaviors which require cognitive computations such as learning the spatiotemporal distribution of food, route planning, statistical inference of food availability, and decision-making [13, 14].

Behavioral, comparative, evolutionary, and genetic studies of wild animals have provided insight into the motivations, behavioral implementations, and genes associated with foraging decisions [5, 15]. Laboratory studies of foraging have provided complementary insight, leveraging a diversity of tools to gain a deeper understanding of the molecular and cellular mechanisms underlying these critical and ubiquitous behaviors. Thus, one approach towards gaining a mechanistic understanding of behavior is to investigate neuroethological hypotheses using common biological model systems. In this review, we argue that the microscopic nematode *Caenorhabditis elegans* is well-suited for studying the molecular and cellular basis of foraging decisions. This one-millimeter-long roundworm boasts a compact nervous system of just 302 neurons with known identity and synaptic connectivity [16, 17]. A myriad of genetic tools, behavioral assays, and neuronal imaging techniques have been developed to take advantage of the species’ quick reproductive cycle, isogeneity, optical transparency, and ease of maintenance [16, 18, 19]. *C. elegans* foraging has been well-studied and, despite the relative simplicity of its nervous system, these animals display robust species-typical behaviors [20] involving learning and memory [21–24], and decision-making [25–30].

Laboratory strains of *C. elegans* were originally isolated from soil samples and were later found to feed on bacteria colonizing decaying organic matter [31–33]. In the laboratory, these animals are usually grown on agar plates containing large patches of the bacteria *Escherichia coli* as a food source [16]. Individuals are generally observed lying on their side moving along the moist agar surface propelled by dorsal-ventral undulations [17, 20, 34–36]. Though *C. elegans* predominantly crawl in a forward direction which can be biased to their ventral or dorsal side resulting in a curved path [37], animals occasionally reorient themselves via stochastic yet stereotyped movements [35, 36, 38]. These simple locomotory movements enable *C. elegans* to control its bearing and navigate an environment [39, 40]. Accordingly, many *C. elegans* behaviors including foraging, mating, and escape responses can be described by an animal’s locomotion and the mechanisms by which underlying neuronal circuits and signaling pathways drive locomotory changes in response to sensory stimuli [20, 41]. *C. elegans* locomotion is coordinated by body-wall muscle cells that receive excitatory cholinergic and inhibitory GABAergic input from dorsal and ventral motor neurons [17, 42]. Upstream of these motor neurons, a set of five pairs of premotor command interneurons receive input from sensory and interneurons and coordinate oscillatory head movements as well as forward and backward motion [17, 43, 44] in response to stimuli across a range of sensory modalities [20, 45]. Stimulus-directed behaviors have thus been extensively studied in *C. elegans* in response to volatile and water-soluble chemicals [37, 38, 46–48], temperature [49, 50], oxygen [51], light [52, 53], electric fields [54, 55], humidity [56], and mechanical perturbation [43, 57]. These studies suggest that *C. elegans* can evaluate an environment for stimuli relevant to foraging and other goal-directed behaviors and use that information to drive decision-making. Here, we present a selective review of *C. elegans* foraging decisions, their known molecular and cellular mechanisms, and future perspectives.

## Food search

A common problem among foragers is how to locate food in an environment. This task is simple when food can be detected at a distance but becomes increasingly difficult when sensory information cannot reliably predict food encounter. Here, we summarize what is known about *C. elegans* food search in two different foraging contexts: (1) environments containing stable and densely distributed food where sensory information reliably predicts food encounter and (2) environments containing only diffuse or variable food where alternative strategies must be adopted to increase the probability of encountering food. Further, we discuss mechanisms for evaluating the availability and edibility of food at a perceived food source and how animals adapt when sensory information conflicts with this evaluation.

### Environments where sensory information predicts food location

For many animals, food search is a difficult, multimodal task, often requiring integration of visual and olfactory cues, though other cues (e.g., gustatory, thermal, magnetic, or anemotactic) may also be leveraged for efficient foraging [58, 59]. For example, mosquitoes utilize multiple sensory modalities to find a host organism. At a distance, a mosquito can detect a host organism’s exhaled CO_2_ carried by the wind and later integrate this information with visual and eventually thermal cues once in closer proximity to the host [60]. In *C. elegans*, numerous sensory cues including chemical molecules and compounds [51, 61], mechanical stimuli [62], and blue light [53] have been implicated in guiding foraging behaviors. Stimulus-evoked behavior in *C. elegans* has been particularly well-studied in the context of responses to volatile (odorants) and water-soluble (tastants) chemoattractants [46] which can indicate the presence of food [61] and prime an animal for more efficient exploitation [59]. Observations of these behaviors show that animals can locomote towards chemical attractants and away from repellants via both randomly- and stimulus-directed movements known, respectively, as klinokinesis and klinotaxis.

**Fig. 1:**
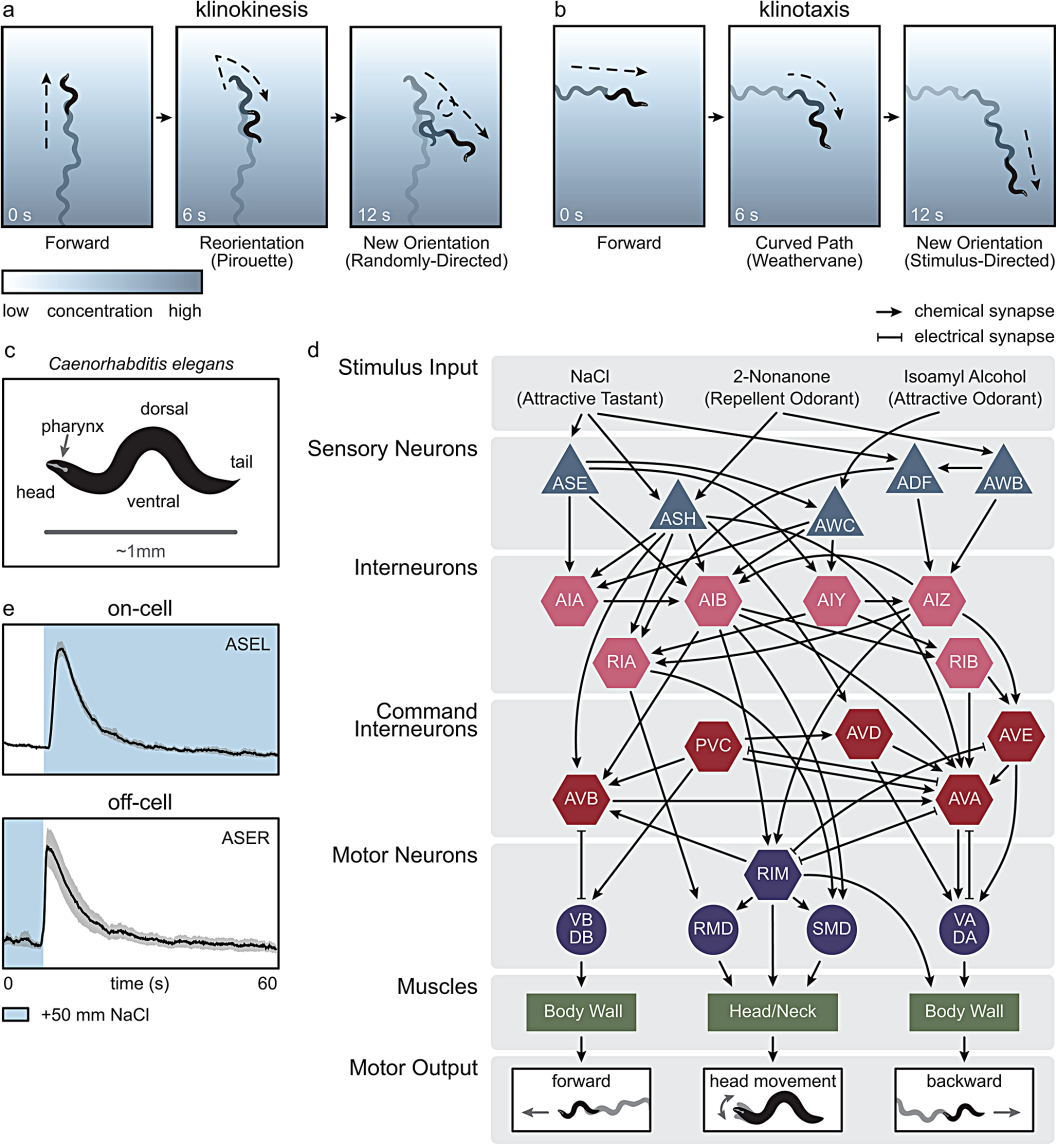
Strategies and mechanisms for food search. (**a**) A representative track of *C. elegans* initiating a reorientation (pirouette) while moving down a concentration gradient, a hallmark of klinokinesis. The animal shown locomotes forward at 0 s, reverses by 6 s, and, by 12 s, reorients its body to face a new direction with continued forward movement. (**b**) A representative track of a curved path (weathervane) initiated during movement perpendicular to a concentration gradient, a process known as klinotaxis. The animal shown locomotes forward at 0 s, initiates a turning bias towards higher concentration by 6 s, and, by 12 s, locomotes up the concentration gradient. (**c**) A simplified anatomical diagram of *C. elegans* showing the head and tail, dorsal and ventral sides, and pharynx. (**d**) Diagram of an example circuit for the encoding of several chemical stimuli within the *C. elegans* nervous system. A subset of sensory neurons (triangles), interneurons (hexagons), motor neurons (circles), and muscle groups (rectangles) known to be involved in the klinotaxis and klinokinesis responses to NaCl, 2-nonanone, and isoamyl alcohol (IAA) are shown. (**e**) Example traces of neuronal activity from calcium imaging experiments of the bilaterally symmetric sensory neuron pair, ASEL and ASER. In response to the addition or removal of NaCl, ASEL and ASER respond as on- and off-cells, respectively. The neuronal activity plotted was taken and adapted from [78]

First described in bacteria, klinokinesis is a common behavioral strategy in which an animal modulates its stochastic reorientation frequency in response to a change in stimulus intensity [63, 64]. For animals moving towards a chemoattractant, suppression of reorientations ensures that they continue in the direction of the stimulus. On the other hand, animals moving away from a chemoattractant should increase their reorientation frequency to redirect movement. In *C. elegans*, klinokinetic reorientations are known as pirouettes (Fig. 1a) [38]. In the presence of a chemoattractant gradient, pirouette frequency is strongly modulated by the rate of change in stimulus concentration over time with pirouettes occurring more frequently when locomoting away from the chemoattractant. Conversely, pirouette frequency has been observed to decrease when locomoting down a chemorepellent gradient [65]. Although these klinokinetic reorientations are mostly non-directional (i.e., the new direction of movement is chosen at random, though with a slight directional bias detected in *C. elegans* [38]), the stimulus-evoked change in frequency eventually results in an overall displacement towards a chemoattractant or away from a chemorepellent.

Klinotaxis is different from klinokinesis in that it describes an animal’s ability to orient their locomotion relative to the direction of the stimulus. In other words, rather than randomly reorienting, animals deterministically steer their forward locomotion in the direction of a chemoattractant. *C. elegans* can navigate towards a chemoattractant via biasing their dorsal-ventral undulations to one side (Fig. 1b) [37, 48]. This klinotactic strategy is known as weathervaning, an analogy to a weathervane orienting in the direction of the wind [38]. When combined, the pirouette and weathervane strategies appear to be sufficient to explain the full extent of chemically-driven behaviors observed in *C. elegans* [37, 66]. Klinokinesis and klinotaxis are common behavioral strategies and have been observed in response to other sensory cues such as temperature in *C. elegans* [49, 50] and other sensory modalities across phyla. For example, *E. coli* adopt a klinokinetic strategy in response to chemical stimuli [64] while *Drosophila* larvae locomote away from a light source, a behavior known as negative phototaxis [67]. Together, these observations suggest that, like many other organisms, *C. elegans* can leverage changes in environmental stimuli to drive goal-directed behaviors such as foraging.

Both the pirouette and weathervane strategies require that *C. elegans* be able to evaluate a spatial chemical gradient in parallel and perpendicular directions to its own locomotion. This presents an interesting challenge for an animal that is one millimeter long and only detects food-relevant chemicals via one functional point in space – the amphid sensory neurons in the head (Fig. 1c) [46, 68]. Without the ability to simultaneously compare the difference in concentration at two spatially separated loci, an animal likely needs to make sequential observations of concentration across time to detect a spatial concentration gradient. Indeed, anatomical, and behavioral observations suggest that *C. elegans* assess a chemical gradient by making comparisons at a single point - the head - through time, thus approximating the rate of change of concentration [38, 46, 69, 70]. This computation is simple for movement up or down the concentration gradient where sequential sampling would reveal a change in concentration. When the chemical gradient is perpendicular to the worm’s own locomotion, rhythmic head sweeping is likely necessary to sample the concentration difference between the dorsal and ventral head positions [37, 66, 71, 72]. In support of this hypothesis, *C. elegans* has been shown to regulate neck bending via integration of motor information and temporal changes in concentration [48]. Altogether, these findings suggest simple sensory mechanisms for driving spatial orientation to a chemical gradient.

The proposed neuronal circuitry underlying these chemically-driven behaviors is relatively simple (Fig. 1d) [66]. Left-right pairs of ciliated chemosensory neurons in the *C. elegans* amphid reliably respond to changes in chemical concentration. For example, avoidance of the repellent 2-nonanone is mediated by the nociceptive neurons ASH and AWB which show a time-differential response to the addition and removal of the odorant, respectively [28]. In response to the attractive odorant isoamyl alcohol, AWC neuronal activity reliably decreases, resulting in suppression of reversals [70, 73–75]. Further, activity in ASE, ASH, and ADF neurons drive chemotactic behavior to tastants (e.g., NaCl) [37, 47, 48, 76–78]. These neurons respond to either the addition or removal of tastants and are known, respectively, as on-cells and off-cells which describes the transient increase in neuronal activation in response to increases or decreases in stimulus concentration (Fig. 1e). The outputs of the on-cells and off-cells converge on the small network of premotor command interneurons (AVA, AVB, PVC, AVD, AVE) that regulate the relative probability of pirouettes and forward locomotion [43, 44]. Integration of sensory and motor information has also been observed further downstream in motor neurons such as SMBD which has been implicated in modulating klinotactic neck bending responses to NaCl [48]. Thus, chemotaxis and chemokinesis behaviors are initiated by sensory neurons which reliably respond to changes in chemical concentration resulting in probabilistic changes in reorientation frequency and dorsal-ventral biases in forward locomotion. Notably, sensory neuron activity does not predict the exact timing of reorientation events. Rather, the activity of the AVA interneurons consistently correlates with both the commencement and termination of reversals [79–81]. Thus, the decision to reorient occurs somewhere in between these sensory and interneurons. Neuronal recordings of the AWC sensory neurons, the AVA command interneurons, and their intermediary synaptic partners (AIB interneurons and RIM inter/motor neurons) reveal a regulatory feedback mechanism controlling the variability in reorientation response [75]. AIB and RIM neurons appear to integrate sensory information with ongoing network state dynamics to produce neuronal and behavioral variability [75, 82]. Altogether, these results suggest that early stages of sensory- and interneuron processing can encode both simple and complex sensorimotor behaviors [75, 82–84]. This early and compact encoding of locomotory signals is not unique to *C. elegans*. For example, neuronal activity in visual cortex has been shown to correlate with the production of facial expressions and body motion in rodents [85]. Further, these results suggest that for behaviors such as foraging which typically persist over long time scales, a single sensory stimulus may not deterministically generate a behavioral response. Rather, integration of sensory information over a longer time window likely provides an ecological advantage.

**Fig. 2:**
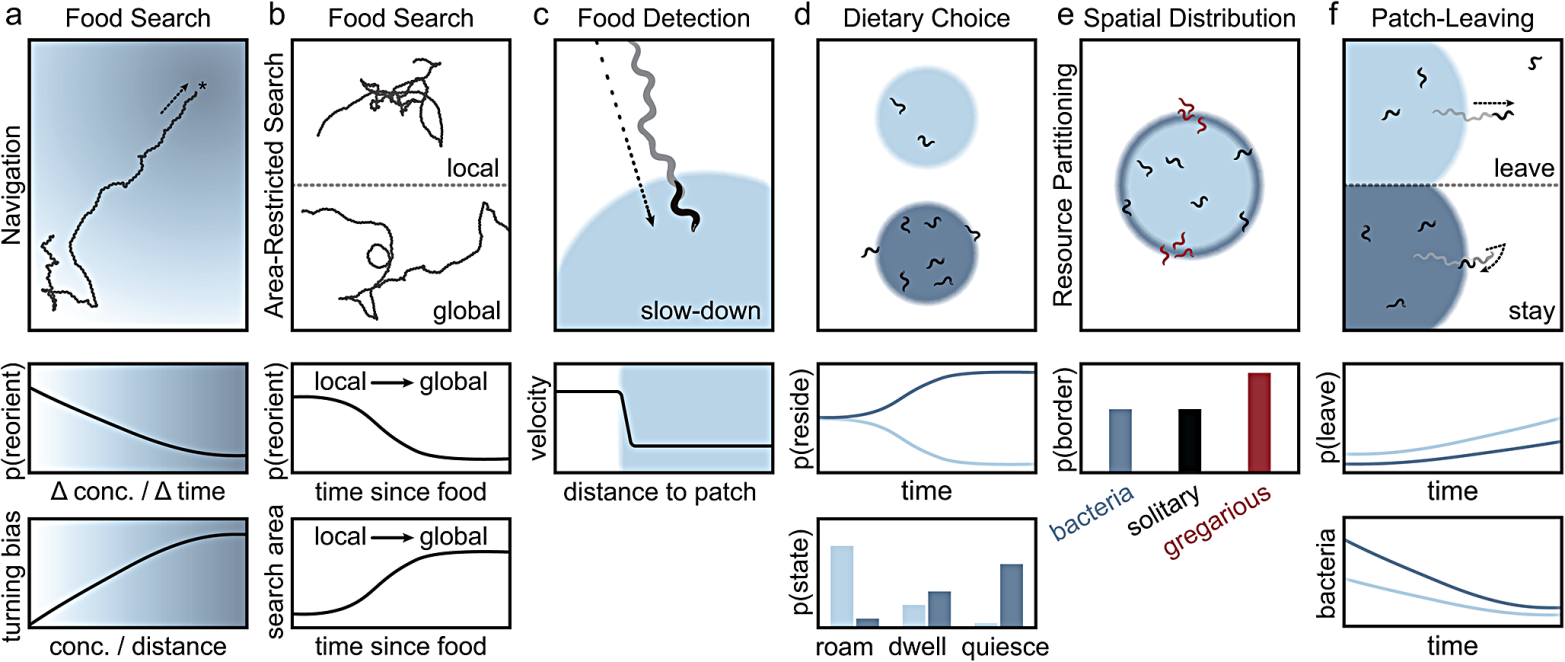
Overview of *C. elegans* foraging decisions. (**a**) *C. elegans* navigate towards an odor source in an environment where a spatial concentration gradient of a food-related odor is present. *C. elegans* odor-guided navigation is characterized by a decrease in the probability of reorientation with increasing rate of change in concentration (klinokinesis) and an increase in the turning bias with an increase in the concentration gradient (klinotaxis). (**b**) *C. elegans* conduct an initial local search followed by a more exploratory global search in an environment where no food-related sensory cues are present. The transition from local to global search is characterized by a decrease in the probability of reorientation and an increase in the search area as a function of time since the animal’s last encounter with food. (**c**) Detection of food is characterized by an abrupt slow-down upon encounter with the patch edge. (**d**) In dietary choice assays, preference for different bacterial food types is often not initially observed as animals must sample bacteria before ascribing it a subjective value. Therefore, the probability of residing on a higher quality patch develops over time. This dietary choice behavior is driven by modulation of exploratory and exploitative behavioral states with animals more likely to be quiescent or dwelling on high quality patches and roaming on low quality patches. (**e**) When animals forage in a group, assessment of within-patch spatial heterogeneity leads to resource partitioning with gregarious strains preferring the dense bacterial patch border and solitary strains distributing themselves proportionately with the bacterial density. (**f**) *C. elegans* may decide to leave or stay upon encounter with the bacterial patch edge. The probability of leaving is higher for less dense and lower quality bacterial patches and further increases as a function of resource depletion as the bacteria is consumed over time

Until recently, most *C. elegans* chemotaxis studies have examined isolated sensorimotor pathways [40, 70, 86] and have revealed largely overlapping circuits consisting of (1) sensory neurons responding to specific stimuli, (2) interneurons coordinating sensory information and network states, and (3) motor neurons driving head and body movements. Recent advances in microscopy have enabled the monitoring and manipulation of large networks of neurons first in immobilized animals [87–89] and subsequently awake and behaving animals [90–92]. Studies leveraging these whole-brain recordings have found that population activity more accurately decodes locomotion than any single neuron and that sensory inputs modulate these continuous global network dynamics [29, 82, 84, 89]. Within this global network, distinct subpopulations of neurons linearly encode specific locomotion dynamics such as velocity and body curvature [82]. As these behavior-correlated neurons do not solely respond to proprioceptive feedback [93], they likely represent low- and high-level motor command signals encoding body postures and longer lasting behavioral states [89]. Further, for some neurons (e.g., AVA), the temporal derivative of activity best correlates with motor dynamics [89], while for other neurons (e.g., AIY), the change in the activity itself predicts behavior [83]. This suggests that neuronal activity at multiple time points is necessary to encode locomotion dynamics [82]. In summary, these studies present a simple mechanism – small neuronal circuits controlling stimulus-evoked reversals and directional bias – that can drive an important and ubiquitous behavior such as food search (Fig. 2a).

### Environments where food location cannot be reliably sensed

While klinokinesis and klinotaxis are efficient foraging strategies in environments with strong spatial gradients, many natural environments contain sparse or patchily distributed food. A forager attempting to detect a shallow or noisy concentration gradient would need to integrate changes in concentration over behaviorally improbable time scales. Therefore, in environments with weak sources of sensory cues, high variability, or unreliable sensory information, an animal must adopt alternative strategies for food search to maximize the probability of encountering randomly located and unpredictably renewed food items. One approach for successful foraging in these sparse environments is to adaptively respond to encounters with food [94]. For example, given the assumption that food is often clumped together in patches, a forager might benefit from remaining in the vicinity of recently encountered food. However, if food is not found, an animal may need to expand food search to a larger area. This type of foraging behavior is known as area-restricted search (ARS) and has been observed in a variety of evolutionarily distinct taxa including protists [95], nematodes [96], insects [97], birds [98], mammals [99], and humans [100, 101]. Like klinokinesis and klinotaxis, area-restricted search is a type of biased random walk with a simple implementation – animals switch between behavioral states of directional exploration (global search) and focused exploitation (local search) in response to resource encounters (Fig. 2b) [102]. Theoretical models of ARS predict that this strategy may be an evolutionary adaptation to foraging in environments where food is patchily distributed in clumps [99, 103, 104].

When moved from a food rich environment to one lacking food (and thus lacking a chemical gradient), *C. elegans* conducts an initial area-restricted, local search via frequent reorientation resulting in minimal travel within the environment [40, 96, 105]. The frequency of reorientation events gradually decreases with increasing time off food, resulting in a transition to a more exploratory period of global search after about 15 minutes. Ecologically, this strategy suggests that animals adaptively broaden their food search as they learn that food is not nearby and focus their search to a more local area when food is detected. Consistent with area-restricted search in other organisms, upon reencountering food, *C. elegans* increases the frequency and magnitude of reorientations [40, 96, 105]. Although *C. elegans* locomotion is dependent on a wide variety of sensory stimuli, only chemical and mechanical sensation of food items appear necessary to transition from global back to local search [62, 96]. Further observations of *C. elegans* foraging reveal that the initial area-restricted search is modulated by prior experience. For example, the size of the bacterial patch an animal experienced immediately preceding food search regulates the reorientation frequency and thus the area explored during local search [24]. Additionally, sustained local search may occur following food-deprivation during larval development [106]. Quantitatively, ARS can be explained by a maximally informative search strategy known as infotaxis, wherein a searcher seeks to continuously maximize information about a stimulus source [107]. An infotaxis model has been used to describe local and global search in *C. elegans* [108]. Altogether, observations of area-restricted search in *C. elegans* suggest that simple heuristics can guide complex behaviors such as food search in an environment with diffuse or variable food.

At cellular and molecular levels, the implementation of ARS in *C. elegans* has been partially elucidated. The local search and dispersal behaviors observed in *C. elegans* ARS are initiated by several ciliated amphid sensory neurons detecting food-related gustatory and olfactory stimuli [40] and additional ciliated cephalic sensory neurons detecting mechanosensory stimuli [96]. Additional studies have suggested a role for numerous other amphid sensory neurons responding to a myriad of polymodal stimuli including odorants, tastants, temperature, oxygen, CO_2_, osmolarity, light, and pheromones as well as mechanical and electrical perturbations [105, 109]. In all cases, these sensory neurons modulate the activity of the locomotory command interneurons directly via classical neurotransmission (e.g., glutamate, acetylcholine, GABA) [40, 73, 96, 105, 109] or indirectly via neuropeptide (e.g., insulin) [110], biogenic amine (i.e., serotonin, octopamine, tyramine, dopamine) [24, 62, 96, 111], and ligand (e.g., TGF-β) [112] signaling pathways. Given the ecological importance of foraging and the range of environmental stimuli that influence locomotion, multiple circuits and signaling pathways likely drive area-restricted search by transducing multisensory information about the presence or absence of food. The specific pathway recruited for a foraging task may be highly contextual, relying on the combination of relevant sensory cues changing in the environment. Despite the diversity in proposed foraging circuits and signaling molecules, these studies collectively show that changes in food-related environmental stimuli are transduced by sensory, inter-, and motor neurons resulting in the regulation of reorientation frequency.

Further understanding of the neurons sufficient to control ARS and the neurons driving the decision to reorient has been limited by the ability to monitor and manipulate neuronal activity over longer timescales. Functional mapping of the neuronal circuits underlying these foraging strategies have been primarily achieved via behavioral analysis of mutants, cell ablation studies, and recording of neuronal activity in immobilized animals. Early studies employing electrophysiological and calcium imaging techniques enabled greater understanding of the dynamics of small networks of contributing neurons but were limited by photobleaching and fictive behavioral outputs due to the restraint of animals for imaging. Recent technological advances have enabled long-term tracking and optogenetic manipulation of the activity of all participating neurons in these behavioral circuits. Leveraging these technologies for answering foraging questions has already revealed important mechanistic insights in *C. elegans* and other organisms and may enable a better understanding of the circuit mechanisms sufficient to drive the decision to reorient. For example, a study monitoring whole-brain neuronal activity at cellular resolution in freely-moving zebrafish larvae found a subpopulation of neurons that encode an ARS-like exploitation state [113]. This study suggests that similar mechanisms for driving and transitioning between persistent behavioral states may have evolved in invertebrate and vertebrate nervous systems and highlights the utility of these advancements in technology. Further, this study and others suggest that goal-directed behaviors such as food search are likely driven by fluctuations in internal states of motivation and arousal [113–115]. Correspondingly, current models describing both ARS and klinokinesis in *C. elegans* suggest a potential encoding of the internal state(s) driving food search wherein transient increases in sensory neuron activity drive probabilistic modulation of reorientation frequency.

### Detection of a food source

Klinokinesis, klinotaxis, and area-restricted search are all goal-directed foraging behaviors which aim to increase the probability of finding food in an environment. However, even when sensory information is reliable, attractive cues may be paired with food that is inedible, toxic, or absent. Therefore, an animal must be able to identify the presence or absence of edible food and respond accordingly. In the case of klinokinesis and klinotaxis behaviors, *C. elegans* reliably locomote towards an environmental chemoattractant even in the absence of food. However, if no food is detected once the animal reaches the odor source, they eventually habituate to the odor and explore other areas of the environment [21]. This habituation response, a form of non-associative learning, has been observed for a variety of chemical and mechanosensory cues [21, 23, 57, 116]. For example, continuous exposure to an odorant results in an odor-specific loss of behavioral response [21], though prolonged exposure to one odorant can facilitate the response to another odorant sensed by the same neuron [116]. This habituation response may be driven by the attenuation of sensory neuron activity [110]. These behavioral observations suggest that *C. elegans* suppress sensitivity to a chemoattractant that is associated with the absence of food to promote continuation of food search. *C. elegans* have evolved additional mechanisms to ensure the continuation of and adaptability of food search when food is not found. Notably, the valence of numerous sensory cues can be altered and sometimes completely reversed during periods of acute food deprivation. For example, after food deprivation, aversion to the *C. elegans*-isolated pheromone ascr#3 is enhanced [117] while aversion to copper, primaquine, and glycerol are attenuated [118, 119]. Further, CO_2_ – a cue emitted by bacteria, predators, competitors, and pathogens – shifts from a repulsive to attractive cue following food deprivation [120]. Valence reversal may indicate a willingness for animals to partake in riskier behavior – prioritizing food search above avoidance of predators and pathogens. These food-deprivation-dependent responses are mediated by signaling from insulin-like peptides from the intestine [117, 119] and several biogenic amines [118, 120] including dopamine and octopamine. Taken together, these findings suggest that neuronal activity can be modulated to promote adaptive food seeking behaviors when sensory information conflicts with food availability and edibility.

Upon discovery of a food source, *C. elegans* display an immediate behavioral response – a drastic slowing of their forward locomotion (Fig. 2c) [27, 62]. This abrupt slowdown upon encounter with the edge of a bacterial patch is likely advantageous for foraging in a patchy environment where a delayed reaction may result in a lost opportunity to exploit. Indeed, animals deficient in serotonergic signaling display delayed slowdown kinetics upon food encounter and, resultantly, are less successful at foraging in a patchy environment [27]. Serotonergic signaling thus appears to confer an exploitation advantage in a complex environment by accelerating decision-making [27]. After detection of a bacterial patch, subsequent evaluation of the bacteria as a suitable source of nutrition and energy is likely an integration of external and internal sensory cues as well as prior experience. Studies of *C. elegans* foraging on (1) food that can be mechanically and chemically sensed but not consumed [121] and (2) food that can be consumed but not sensed, suggest that the internal perception of food via post-ingestive feedback is necessary to induce exploitation while external sensory perception promotes exploration [86, 122, 123]. In further support of this role for post-ingestive feedback, a serotonergic neuron (NSM) has been implicated as an enteric sensory neuron, acutely detecting food ingestion via acid-sensing ion channels (ASICs) [124]. Collectively, these results suggest that abrupt slowdown facilitates detection of a food encounter via external and internal cues.

Altogether, studies of food search suggest that *C. elegans* use simple sensory-evoked search strategies – klinokinesis, klinotaxis, and area-restricted search – to increase the probability of food encounter. Animals subsequently evaluate whether the available sensory information is associated with the presence or absence of food and adaptively modulate their behavior to either resume food search or transition to a new foraging behavior.

## Dietary choice

While the hardest task for many foragers is locating a food source, for others, food encounters are frequent due to a dense distribution of food in the environment. In either case, once food is located, an animal must evaluate individual items or patches of food and select those that maximize fitness. Here, we summarize what is known about *C. elegans* dietary choice in two different foraging contexts: (1) individual foragers evaluating a food type for inclusion in their diet, and (2) groups of foragers competing for food items within a patch.

### Individual foraging

Foraging theory predicts that an encounter with food does not necessarily result in exploitation; rather, an animal may choose to accept or reject a food item upon encounter. Thus, a foraging animal must consider what types of food to include in its diet. Within foraging theory, a class of models known as optimal diet theory (ODT) has sought to rationalize this foraging decision [1, 2]. These models have since been extended to include optimal patch choice, an analogous problem for patches of food [4]. According to the original formulation of ODT, an optimal diet is one which maximizes the net rate of energy or mass intake within a fine-grained, patchy environment [4]. In this model, food types are ranked by their associated value measured in calories (or mass) per time spent searching and handling. Food types are included in the diet as long as their inclusion does not decrease the net rate of food intake [2]. In other words, an optimal forager will specialize its diet, only consuming the highest quality food items when those food items are sufficiently abundant. Canonical ODT bases the dietary choice decision only on the relative value and abundance of food types. However, a real-world forager may also be concerned with other constraints such as nutrients, toxins, predation, hedonic value, and thermoregulation [5]. For example, food can be either rewarding (e.g., when energy gained after ingestion provides reinforcement) or aversive (e.g., when food is poisonous). Additionally, for many animals, diet selection involves balancing nutrients for optimal growth to the extent that nutrient-deficiencies can lead to an increased appetite for a specific nutrient. Further, mammals can learn to choose the food source solely based on its nutritional contents even when no taste cues are available [125]. Studies of *C. elegans* foraging have led to important insights into how animals integrate several food-related cues to drive dietary choice behavior.

While foraging on food, *C. elegans* display several alternative behavioral states – roaming, dwelling, and quiescence [112, 122]. The time spent in each of these states is dependent on food quality and quantity as well as prior experience. Comparably, on-food foraging strategies of *Drosophila* have been shown to vary depending on the food quantity present in the environment [126]. When grown on OP50, the common lab strain of *E. coli*, *C. elegans* typically spend 80% of their time in a dwelling state characterized by slow movements with frequent alternation between backward and forward motion [122]. The other 20% of time is typically spent in a state of straight, sinusoidal forward movement known as roaming. Bouts of roaming often last one or two minutes and are interspersed with longer-lasting – 5 to 10 min – dwelling periods [127]. With increasing food density and nutritional quality, *C. elegans* further decrease their on-food speed and spend more time dwelling [86, 128]. Further, in the presence of food, *C. elegans* increase the rate that their pharynx pumps food into their intestine [112, 129, 130]. However, on high-quality bacteria (e.g., *E. coli* HB101 or *Comomonas* sp.) wild-type *C. elegans* are quiescent 90% of the time, displaying a complete cessation of both pharyngeal pumping and locomotion [112]. Quiescence is likely a result of satiety and is dependent upon food quality, nutritional signals from the intestine, and prior feeding history. A relatively small difference in bacterial quality can result in the presence or absence of the sleep-like quiescent behavioral state [112, 128]. Altogether, these results suggest that *C. elegans* can precisely vary the time allocated to each activity phase in a nutritional-, concentration-, and experience-dependent manner while foraging on a bacterial patch.

In the wild, *C. elegans* are exposed to and feed upon a diversity of bacterial types (~ 2,400 operational taxonomic units) with varying biological value and pathogenicity [131]. The value or quality of these bacterial strains can be defined by their ability to support animal growth [128]. Some results suggest that, for *C. elegans*, bacterial quality is most highly correlated with bacterial density [132] and size (i.e., smaller bacteria better support *C. elegans* growth) [130] as opposed to other characteristics such as toxicity. Therefore, detection of food quality is likely most important for young larvae with small pharynges. Further, although *C. elegans* is able to persist in a wide range of microbial communities [131], they strongly prefer bacterial species that support animal growth [128]. When given a choice of bacterial food types, they behave in a manner consistent with optimal diet theory [1, 2] and rational choice theory (Fig. 2d) [133]. Preference for higher quality bacteria is independent of innate chemotactic biases and likely requires post-ingestive feedback after sampling the food choices. Accordingly, food type preferences are internally consistent (i.e., if bacteria A > B and B > C, then A > C) [134] while innate chemotactic preferences are only generally consistent [135, 136]. Consistency of choice suggests an underlying representation of subjective value and is the hallmark of a rational decision-maker [133]. For *C. elegans*, the subjective value or utility of bacterial species must be learned, a process requiring dopaminergic signaling [134]. These results suggest that *C. elegans* displays bounded rationality wherein non-optimal decision-making can be explained by inherent biological limitations [135, 136]. Altogether, these studies suggest that *C. elegans* can learn, remember, and compare the quality of bacterial species and use that information to maximize foraging.

In dietary choice assays where animals are presented with more than one food option, *C. elegans* modulate their time spent in dwelling and roaming states in order to spend more time foraging on higher-quality bacterial patches [128]. Dwelling likely ensures maximal food intake while roaming enables exploration of new resources with the risk of missing the opportunity to fully exploit the local food. Evidence suggests that this foraging strategy may be a memory-dependent behavior that relies on both the animal’s current environment and the environments it has experienced previously. For example, worms in a dietary choice assay are more likely to reject mediocre food if they previously experienced a higher-quality bacterial species [128, 134]. Further, animals that have been food-deprived display a sustained enhancement in time spent dwelling even after reencountering food [62, 86]. Sudden food encounter after prolonged food deprivation triggers quiescence [112]. Altogether, these studies suggest that *C. elegans* display persistent, long-lasting behavioral states during foraging on food and that transitions between these states are likely driven by external and internal sensory cues and modulated by prior experience.

Studies in *C. elegans* have revealed some insights into the neuronal and molecular pathways underlying the stability and transitions between foraging states and highlight the importance of neuromodulation in driving persistent behavioral states [137]. In *C. elegans*, most neurons communicate via graded synaptic transmission [138], though calcium-mediated action potentials [139, 140] and plateau potentials [141] have been observed. In addition to classical neurotransmission, regulation of the *C. elegans* nervous system is highly dependent on signaling of neuropeptides [142], biogenic amines [143], and intracellular ligands, many of which share considerable homology with other invertebrate and vertebrate systems and play a role in modulating behaviors. For example, the biogenic amines serotonin and dopamine promote food-related behaviors such as eating, egg-laying, and slowed locomotion [62, 111, 144], while octopamine – the invertebrate homolog of norepinephrine – and tyramine act antagonistically, mimicking the absence of food [25, 111, 145]. Further, neuropeptides (e.g., vasopressin, neuromedin U, orexin, oxytocin) are well known to modulate persistent behavioral states (e.g., hunger, thirst, arousal, sleep) in *C. elegans* [110, 142, 146] and other animals including insects [147], crustaceans [148], and mammals [149]. In the context of on-food behaviors in *C. elegans*, serotonin signaling promotes dwelling [122, 127] while the neuropeptide PDF and insulin promote roaming [86, 127]. Additionally, intracellular signaling via the cGMP-dependent protein kinase EGL-4 and the ligand TGF-β modulate roaming, dwelling, and quiescence [86, 112, 123]. Together, these results suggest that neuropeptides, biogenic amines, and intracellular signaling molecules play an important role in modulating roaming and dwelling states on a bacterial patch. Future research leveraging advancements in microscopy to monitor and manipulate long-timescale behaviors [150, 151] will lead to further insights into how these molecular pathways interact and coordinate persistent behavioral states.

### Collective foraging

Models of optimal patch choice generally make a simplifying assumption that food items within patches are equally distributed. However, for many animals, food is heterogeneously distributed within a patch. Therefore, consideration of within-patch spatial heterogeneity may be beneficial for a forager, especially when competing for food with other individuals. For *C. elegans* in the laboratory, bacterial patches grown on agar plates are denser at the patch edge where actively proliferating bacteria are concentrated [152, 153]. Individuals of the common lab strain N2 distribute themselves throughout the patch in a manner proportionate to the bacterial density and are therefore considered solitary foragers (Fig. 2e) [153]. On the other hand, most wild strains of *C. elegans* are gregarious foragers which aggregate at the edge of a bacterial patch, a behavior known as bordering [154]. Resultantly, groups of gregarious animals disproportionately consume bacteria at the edge of the bacterial patch while groups of solitary foragers maintain a patch’s relative spatial density. When mixed, these strains show spatial resource partitioning with gregarious worms residing at the border while solitary individuals forage in the center of the bacterial patch [153]. Solitary foragers outcompete gregarious foragers on a large patch of bacteria, but in environments containing much smaller bacterial patches, animals perform equally well. The behavior of solitary foragers is seemingly consistent with both the matching law [155] and an ideal free distribution (IFD) [156], theories that predict that either individuals or groups of animals, respectively, will distribute themselves across a habitat in a manner proportionate to the resources available. This observation suggests that solitary strains have evolved to optimize foraging on a continuous food environment while gregarious strains display greater dispersal propensity, a trait advantageous for a fragmented or patchy environment. Notably, this hypothesis is well-aligned with the domestication of solitary strains in laboratory conditions [157] while gregarious strains evolved in a boom-and-bust environment in the soil [33]. Altogether, these results suggest that, even within a patch, *C. elegans* are capable of modulating where and what they are foraging on. This spatial resource partitioning is ecologically advantageous when foraging in competition with inter- or intra-specific individuals.

The phenotypic difference between gregarious and solitary strains is a result of a single amino acid change in the G-protein coupled receptor NPR-1 which acts via an oxygen-sensing pathway [153, 154]. The gregarious *npr-1* allele confers a preference for hypoxic environments such as the patch edge where bacterial respiration lowers oxygen levels [153, 154]. A related phenotype is seen in the fruit fly (*Drosophila melanogaster*) larvae where variation in the *foraging* gene, which encodes a cGMP-dependent protein kinase, confers a rover or sitter phenotype [158]. Like gregarious nematodes, the rover larvae display higher dispersal propensity in a patchy food environment. Further, a *foraging*-related cGMP-dependent kinase affects foraging behaviors in other animals including ants, honeybees, and nematodes [112, 159, 160], suggesting that diverse animals share molecular mechanisms for behavioral regulation. In summary, *C. elegans* has evolved a unique mechanism for spatial resource partitioning which may confer an advantage during collective foraging in varied habitat types.

## Patch-leaving

In environments where food is clumped in patches, detection, and acceptance of one food item generally leads to frequent subsequent encounters with additional items. However, in many cases, a patch will exhibit diminishing returns as food is consumed at a greater rate than it is replenished. Therefore, a fundamental question in behavioral ecology is how a forager decides how long to exploit a patch before moving on to explore potentially better options [5]. Foraging animals must be able to balance the benefits of remaining in their current patch despite diminishing returns against the potential lost opportunity of finding a higher-quality food patch elsewhere. Patch foraging is thus a sequential decision-making problem that requires the organism to optimize a trade-off between exploration and exploitation. The Marginal Value Theorem (MVT) has been used to describe this tradeoff by defining the relationship between the rate of food intake on a given patch and the optimal amount of time an animal should reside on that patch [161]. This simple model predicts that patches should be left when the marginal capture rate equals the long-term average rate of energy intake [5]. In other words, the optimal time for an animal to leave a food patch occurs when the local resource level falls below the average level in the entire habitat. A broad diversity of animals across phyla exhibit patch-leaving behaviors that are well-approximated by the MVT [5].

Once on a high-quality food patch, well-fed *C. elegans* rarely leave (Fig. 2f). However, the frequency of patch-leaving has been shown to increase with declining food quality and quantity, when bacteria is inedible, and when feeding is impaired [25, 128, 162, 163]. Additionally, patch-leaving events can be modulated by an animal’s metabolic status as well as the presence of pathogens [164, 165], chemorepellents [166], predators [167, 168], and varying levels of environmental O_2_ and CO_2_ [162, 169]. In some wild isolates, patch-leaving frequency is also suppressed by early-life food deprivation [106]. Further, the decision to leave a patch of food is linked to the animal’s arousal state as leaving occurs probabilistically during the high arousal roaming state, but is suppressed during dwelling [30]. These results suggest that patch-leaving is strongly modulated by a variety of internal and external sensory cues over a range of behavioral timescales.

Like most natural behaviors, the decision to leave a food patch is regulated by multiple neuronal and signaling pathways. The general propensity for *C. elegans* to remain on a food patch of suitable quality is dependent on the invertebrate noradrenaline-like neurotransmitters, tyramine and octopamine (*tyra-3*) [25] and the neuropeptide receptor NPR-1 which regulates food-related behaviors such as aggregation [154] and dispersal [153]. Most wild-isolates contain a polymorphism in the *npr-1* gene which results in higher incidences of food-leaving, an effect mediated by sensation of ambient O_2_ [162]. The N2 *npr-1* allele is epistatic to *tyra-3* and this relationship is environmentally dependent [25]. The sensory neurons that express *tyra-3* (ASK, BAG, and others) also detect food related cues suggesting that these neurons integrate external cues with internal arousal states and that different *tyra-3* alleles confer differential sensitivity to these arousal states [25]. Furthermore, insulin-like neuropeptides and the TGF-β-like ligand DAF-7 appear to play a role in promoting adaptive food-leaving behavior [162]. Additionally, animals with loss-of-function mutations in genes encoding subunits of cGMP-gated channels (*tax-4* or *tax-2*) show reduced probability of patch leaving, while animals with mutations in genes encoding transient receptor potential V-like ion channels (*osm-9* or *ocr-2*) display increased food leaving [162]. The cGMP-gated transduction channel TAX-4 also appears to be involved in the coupling of roaming and patch-leaving dynamics [30]. Altogether, these results suggest that regulation of patch-leaving behaviors is complex and highly correlated with global behavioral states such as roaming and dwelling.

## Considerations

### Towards a more ethological approach

**Fig. 3:**
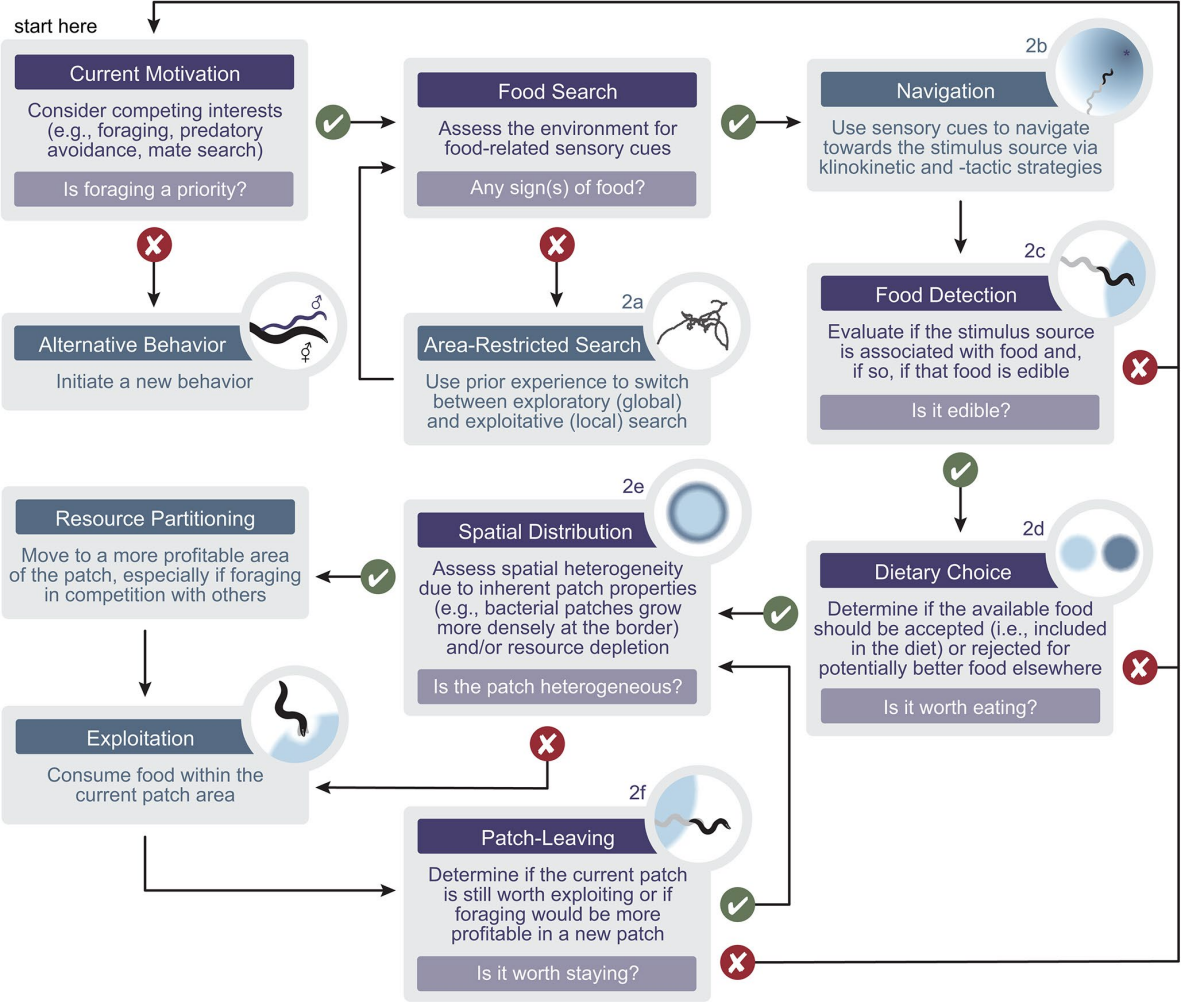
Foraging decisions are often cyclical. A decision tree of the *C. elegans* foraging decisions described in this review is shown. Corresponding elements to Fig. 2 are indicated. Actions regarding alternative behaviors, area-restricted search, navigation, resource partitioning, and exploitation are described in blue-hued boxes. Decisions regarding an animal’s current motivation, food search, food detection, dietary choice, spatial distribution, and patch-leaving are described in purple-hued boxes. Decisions result in binarized consequences (yes – green check; no – red X) leading to the next decision or action

Our understanding of the neuronal mechanisms driving goal-oriented behaviors such as foraging has been historically limited by simplified laboratory conditions and experimental designs which primarily seek to correlate neuronal activity with behavioral observations [170]. Although this strategy has led to important mechanistic insights, a more complete understanding of how the nervous system has evolved to drive behavior requires a holistic view of the motivations and implementations of natural behaviors during ecologically relevant tasks (Fig. 3). In the case of foraging, we must consider an animal’s internal state, biotic and abiotic environment, prior experience, and alternative motivations (e.g., reproduction and predatory avoidance) that may conflict with their ability to optimize foraging. Further, we must be careful not to dissect our study of animal behavior into ethologically irrelevant fragments. Just as the meaning of a sentence is lost by viewing each letter in isolation, our neuroethological understanding of foraging may be limited by studying foraging decisions one at a time. Consideration of the cyclical nature of foraging (e.g., search, evaluation, consumption, then search again) [5] and neuronal dynamics that match the longer timescale of these cycles may be critical to our understanding of the nervous system’s role in driving persistent states of animal behavior.

Many recent studies in a variety of model systems from single-celled microorganisms to vertebrates have demonstrated that ecologically focused environmental enrichment permits identification of novel behaviors and gene functions [171–173]. For example, *C. elegans* foraging has been almost exclusively studied in the laboratory on agar plates where individuals generally lie on their side and move only in two dimensions [17, 20, 34–36]. However, in the wild, *C. elegans* live in the soil and are free to move in three dimensions [31–33, 131]. A recent study found that when locomoting in a three-dimensional substrate, populations of dauer *C. elegans* exhibit complex nictation behaviors including jumping which is rarely observed in animals residing on agar plates [174]. Further consideration of abiotic factors may be important for parsing underlying fitness variation under natural conditions as wild *C. elegans* isolates display variable preferences for temperature and humidity [175, 176]. Beyond the abiotic environment, many studies have highlighted the complex interactions between microbial communities and their host species [177, 178]. *C. elegans* are found worldwide in soil samples containing decomposing plant material which provides an ample bacterial food source for the nematode [31–33, 131]. However, most laboratory *C. elegans* foraging experiments have sampled a relatively small number of bacterial species compared with the vast diversity of species (~ 2,400 operational taxonomic units) available in its natural habitat [131]. *C. elegans* has emerged as a powerful model system for studying host-microbiome interactions [179, 180] with recent efforts supporting a more ecologically-relevant model of *C. elegans* microbiota (CeMbio) [181]. Further, large patches of densely seeded bacteria have primarily been used in experiments assessing *C. elegans* foraging. However, wild nematodes experience a boom-and-bust environment [33] that may be more consistent with sparse, patchily distributed bacteria [27]. Thus, just as quiescence is only observed on high quality bacterial patches [112], additional behavioral states may be discovered when assessing foraging in patchy environments. Future studies must continue to seek more naturalistic observations of *C. elegans* behavior to achieve a more complete cellular, molecular, and genetic understanding of how the nervous system has evolved to drive animal behavior.

Artificial laboratory constraints extend beyond abiotic and biotic environmental factors. Notably, the standard wild-type *C. elegans* strain, N2, displays markedly different behavior from most natural isolates which form aggregates at the border of a bacterial patch where food is thickest [154]. This phenotypic variation is due to a gain-of-function mutation in the neuropeptide receptor *npr-1* gene in the N2 strain during laboratory domestication which confers a solitary foraging phenotype [154, 157, 182]. The solitary and gregarious foraging phenotypes appear to be differentially advantageous in environments with varying food distribution [153, 183]. These considerations highlight that while many important insights have been gained via simplified studies of animal behavior, we have been limited to understanding what the nervous system is capable of rather than what it has evolved to do. Incorporating biologically relevant parameters will enable a deeper understanding of conserved principles of decision-making and other cognitive processes that have come about via natural selection.

Further, it is important to consider that foraging does not occur in isolation. Animals must integrate complex information about their external environment and internal state and may have to manage competing motivations such as mating, predation, and competition. *C. elegans* shares its microhabitat with arthropods, microorganisms (i.e., bacteria and fungi), and invertebrates including other nematodes [33]. Thus, like all animals, *C. elegans* must weigh foraging against other needs such as avoidance of pathogens and predators. For example, *C. elegans* interacts with obligate and non-obligate parasites such as fungi, microsporidia, bacteria, and viruses [33]. Possible natural predators include small arthropods and other nematodes such as *Pristionchus sp*. In studies where *C. elegans* is paired with a predatory nematode, *C. elegans* learns to avoid a predator-occupied bacterial patch even after the predator has been removed and at the expense of reduced survival of progeny [167, 168]. However, just as foraging optimization can be deprioritized when predatory risk is high, avoidance of some aversive compounds (e.g., copper, primaquine, glycerol) is attenuated when successful foraging is critical (e.g., when animals are food-deprived) [118, 119]. Thus, the behavior of *C. elegans* foraging in the wild where predators and pathogens cohabitate may appear very different from what has been observed in the laboratory. Further, in addition to the avoidance of harmful agents, *C. elegans* must weigh foraging goals against reproductive success. For example, compared with hermaphrodites, male *C. elegans* are much more likely to leave food patches in search of potential mates [184, 185]; though, consequently, food-deprived males are less efficient at mating compared to their well-fed counterparts [121]. Additionally, *C. elegans* foraging is affected by the density of individuals present via pheromone signaling [186]. Together, these results suggest that the motivation for foraging must be weighed against other priorities that affect an animal’s fitness such as mating and risk avoidance. Further, they highlight the need for more ethological studies representing the complex needs of animals in the wild to gain a deeper understanding of decision-making.

### Leveraging methodological advancements

Most studies of *C. elegans* behavior have used genetic mutants and cell ablations to parse the underlying neuronal and molecular pathways. These studies have thus been limited in their ability to parse complex interactions between neuronal circuits and signaling pathways and in their ability to monitor and manipulate persistent, long-lasting behavioral states. In recent years, technological advances have led to exciting new opportunities for understanding animal behavior from macro- to microscales.

At the organismal scale, significant strides have been made towards development of assays that monitor and manipulate *C. elegans* behavior. For example, the employment of microfluidics in *C. elegans* behavioral and neuronal imaging experiments has enabled precise control of the spatial and temporal dynamics of chemical stimuli [74, 79, 187–190] as well as high-throughput automation of quantitative analyses of neuronal activity and behavior [80, 81, 188, 189, 191–193]. Additional advancements in device fabrication, imaging, and analysis methods have ameliorated longitudinal studies of *C. elegans* behavior [150, 151, 194–197].

At the cellular scale, advancements in microscopy have enabled imaging of the activity of large networks of neurons in both immobilized [89] and freely-moving animals [18, 29, 82, 88, 90–92]. Additionally, development of a neuronal landmark strain (NeuroPAL) providing unambiguous identification of neuronal identity enables integration of structural and functional connectivity for whole-brain imaging applications [198]. Further, employment of opto-, chemo-, and sonogenetic techniques has enabled precise manipulation of neuronal activity in freely moving animals [199–202]. Studies employing these techniques have led to important insights into how global brain states control persistent and flexible behavioral states such as foraging. Further research in the field seeks to identify circuit mechanisms that couple long-term behavioral states with short-term decision-making and motor actions in foraging and other goal-directed behaviors.

At the molecular scale, numerous tools have been developed for cataloging and manipulating genes, RNA, proteins, peptides, and metabolites. For example, the *C. elegans* Neuronal Gene Expression Map & Network (CeNGEN) has established a comprehensive gene expression atlas of the entire nervous system at single-cell resolution [203] enabling investigation of the fundamental mechanisms that generate neuronal diversity including regulation of gene expression, alternative splicing, and miRNA function [204]. Researchers have also achieved system-wide mapping of peptide-receptor interactions [205]. Additional advancements in genetic tools have enabled precise spatial and temporal control of gene and protein expression. A variety of tools have been developed for stage- and tissue-specific expression [206–209] and depletion [210–214] of genes and proteins. Further, significant strides in metabolomics have revealed a connection between individual variation and metabolism in *C. elegans* [215]. Altogether, these advancements highlight the utility of *C. elegans* as a model system and promise a deeper understanding of how animal behavior arises from complex mechanisms at the level of neuronal circuits, gene expression, signaling molecules, and host-microbiome interactions.

## Summary

Foraging is a complex series of behaviors requiring numerous decisions that integrate external, internal, and contextual cues across a range of timescales. Behavioral and mechanistic studies of foraging in *C. elegans* have led to important insights into how goal-directed behaviors are generated. The ability to precisely manipulate *C. elegans* genetics and behavior enables detailed observation of foraging decisions and dissection of the underlying neuronal circuits and signaling pathways. Studies have found that small modules of interconnected neurons process multimodal sensory information and coordinate foraging behaviors, though more global neuronal activity patterns appear to be associated with persistent behavioral states. Maintenance of and transitions between these persistent behavioral states are likely driven by a combination of classical neurotransmitters, neuropeptides, biogenic amines, and intracellular signaling molecules. We suggest that future studies should leverage more ethological approaches and advancements in technology which promise new opportunities to understand the mechanisms underlying decision-making and persistent and flexible behavioral states.

## Data Availability

Not applicable.
